# Associations of the Zhejiang University (ZJU) index with reproductive outcomes in women with polyendocrine metabolic ovarian syndrome: a *post-hoc* analysis

**DOI:** 10.3389/fnut.2026.1892813

**Published:** 2026-08-24

**Authors:** Yang Liu, Yi Gong, Hang Ge, Mengyi Zhu, Hong Yu, Jiaxing Feng, Xinyu Hu, Hui He, Jingshu Gao, Xiaoke Wu

**Affiliations:** 1 The First Affiliated Hospital of Zhejiang Chinese Medical University (Zhejiang Provincial Hospital of Chinese Medicine), Hangzhou, China; 2 Zhejiang Chinese Medical University, Hangzhou, China; 3 Beilun District People's Hospital, Beilun Branch of the First Affiliated Hospital of Zhejiang University, Ningbo, China; 4 Department of Obstetrics and Gynecology, First Affiliated Hospital, Heilongjiang University of Chinese Medicine, Harbin, China; 5 Reproductive Medicine Center, First Affiliated Hospital of Zhengzhou University, Zhengzhou, China

**Keywords:** BMI—body mass index, metabolism, polyendocrine metabolic ovarian syndrome, reproductive outcomes, ZJU index

## Abstract

**Purpose:**

The Zhejiang University (ZJU) index is a novel composite metabolic score incorporating body mass index, fasting glucose, triglycerides, and the alanine aminotransferase/aspartate aminotransferase ratio. This study aimed to evaluate whether baseline ZJU index is associated with reproductive outcomes in women with polyendocrine metabolic ovarian syndrome (PMOS) undergoing fertility treatment.

**Methods:**

This *post-hoc* analysis is based on the Polycystic Ovary Syndrome Acupuncture and Clomiphene Trial (PCOSAct). Participants were stratified into tertiles based on baseline ZJU index levels. Logistic regression models estimated odds ratios (ORs) and 95% confidence intervals (CIs) for ovulation, conception, clinical pregnancy, and live birth, adjusting for potential confounders. Restricted cubic spline (RCS) analyses assessed nonlinear associations, and sensitivity analyses evaluated the robustness of the findings.

**Results:**

Women in the highest ZJU tertile had significantly lower rates of ovulation, conception, clinical pregnancy, and live birth compared with the lowest tertile (all P < 0.05). In the primary adjusted model (Model 2), each one-unit increase in ZJU was associated with reduced odds of ovulation (OR 0.94, 95% CI 0.91–0.97), conception (OR 0.96, 95% CI 0.93–0.99), clinical pregnancy (OR 0.95, 95% CI 0.92–0.98), and live birth (OR 0.95, 95% CI 0.92–0.99). No significant non–linearity was observed (all *P* for non-linearity > 0.05). For the continuous ZJU associations, point-estimate E-values ranged from 1.17 to 1.22, and the corresponding confidence-limit E-values ranged from 1.09 to 1.15, indicating limited robustness to unmeasured confounding. Associations between the ZJU index and reproductive outcomes remained consistent across sensitivity analyses.

**Conclusion:**

A higher baseline ZJU index was associated with poorer reproductive outcomes in women with PMOS undergoing fertility treatment. The ZJU index may serve as a metabolic marker associated with reproductive outcomes in women with PMOS. However, its ability to predict individual reproductive outcomes and any clinically applicable threshold require further evaluation in dedicated prediction studies with external validation.

## 1 Introduction

Polycystic ovary syndrome (PCOS) is the most common endocrine disorder among women of reproductive age, affecting an estimated 5–20% of this population worldwide and accounting for up to 80% of anovulatory infertility (1, 2). However, the landscape of terminology in this field is evolving. The 2023 international evidence-based guidelines have introduced the term polyendocrine metabolic ovarian syndrome (PMOS) to replace the traditional PCOS nomenclature, as PMOS more accurately reflects the core metabolic pathophysiology of this condition and aligns diagnostic criteria with metabolic risk stratification. The global burden of PMOS has been rising, with studies reporting increases in both its incidence and prevalence over recent decades (3). The pathophysiology of PMOS is complex and multifactorial; insulin resistance (IR) and consequent hyperinsulinemia are recognized as central features that exacerbate hyperandrogenism and underlie many of its associated metabolic and reproductive dysfunctions (4, 5). Consequently, women with PMOS face not only impaired fertility but also a significantly elevated risk of adverse pregnancy outcomes (6, 7).

The current landscape of predictive markers for reproductive outcomes in PMOS is dominated by anthropometric and hormonal indices, yet these traditional metrics often fail to capture the full spectrum of metabolic dysfunction inherent to the syndrome (8). Recent research has highlighted the ZJU index, a novel composite score derived from body mass index (BMI), fasting plasma glucose (FPG), triglycerides (TG), and the serum alanine aminotransferase (ALT) to aspartate aminotransferase (AST) ratio. Originally developed by Wang et al. (9) as a screening tool for non-alcoholic fatty liver disease (NAFLD) in the Chinese population, the ZJU index has demonstrated superior diagnostic accuracy compared with other established indices, such as the fatty liver index and hepatic steatosis index, achieving an area under the receiver operating characteristic curve (AUROC) of 0.925 (10, 11). Subsequent research has substantially expanded the clinical utility of the ZJU index. The index is a useful indicator of IR in the general Chinese population (12). Another study linked a higher ZJU index to an increased risk of infertility among reproductive-aged women in the United States (13). Collectively, these studies position the ZJU index as an integrative biomarker of systemic metabolic health, with clear relevance to the metabolic disturbances central to PMOS.

Despite the growing body of research on the ZJU index, its precise relationship with reproductive outcomes in women diagnosed with PMOS remains to be thoroughly investigated. To address this critical gap in the existing literature, our study was designed to elucidate the associations between baseline ZJU index and key reproductive endpoints, thereby providing robust evidence to bridge this knowledge gap.

## 2 Methods

### 2.1 Study population and design

**Figure 1:**
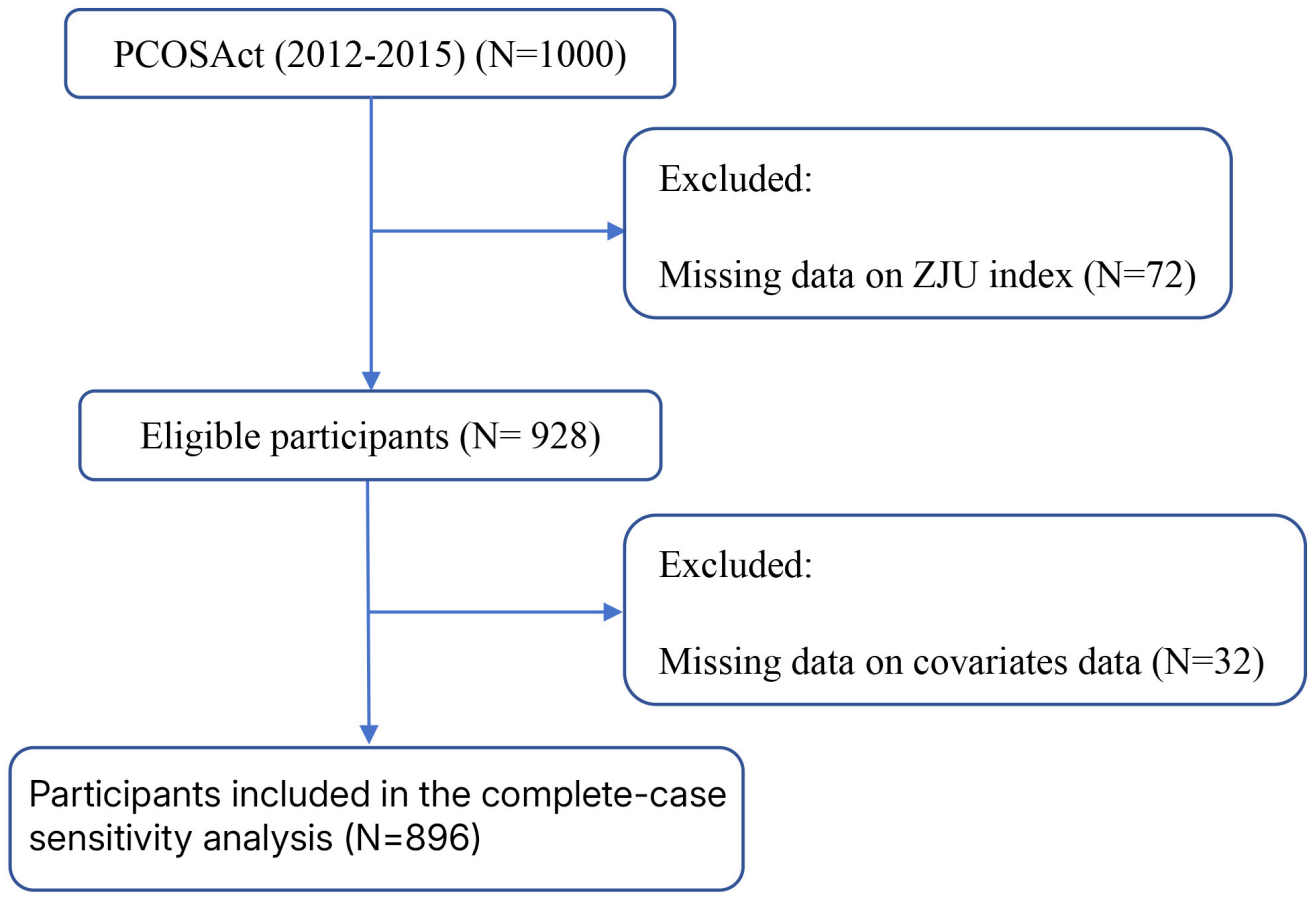
Flow chart of study participants.

Data for this *post-hoc* analysis were obtained from the Polycystic Ovary Syndrome Acupuncture and Clomiphene Trial (PCOSAct), a prospective randomized controlled trial published in JAMA (2017;317:2502-14) (14). This trial is registered on ClinicalTrials.gov (NCT01573858) and Chictr.org.cn (ChiCTR-TRC-12002081), with the registration date set as July 6, 2012 (15). All methods were conducted in accordance with the relevant guidelines and regulations. The research conducted in this study received approval from the Ethics Committee of the First Affiliated Hospital of Heilongjiang University of Chinese Medicine (2010HZYLL-010). Eligible subjects were Chinese with PMOS-related anovulatory infertility and were randomly assigned to one of four groups for four consecutive treatment cycles: (1) CC with active acupuncture, (2) placebo with active acupuncture, (3) CC with control acupuncture, or (4) placebo with control acupuncture, receiving therapy for four consecutive menstrual cycles. All 928 participants with sufficient data to calculate the ZJU index and ascertain the reproductive outcomes were included in the primary analyses. Among them, 896 participants had complete data for all Model 3 covariates and were included in the extended complete-case sensitivity analysis (Figure 1).

### 2.2 Data collection

#### 2.2.1 Baseline evaluation

Demographic and clinical information was collected using a standardized questionnaire. Anthropometric measurements, including age, body weight, height, and waist circumference (WC), were performed by trained nurses according to standard protocols. BMI was calculated as weight in kilograms divided by the square of height in meters (kg/m^2^). Systolic and diastolic blood pressures (SBP and DBP) were measured three times on the right arm after a 5-min rest using an automated electronic device, and the average value was used for analysis. Venous blood samples were obtained after an overnight fast of at least 8 h. Serum levels of luteinizing hormone (LH), follicle-stimulating hormone (FSH), estradiol (E_2_), progesterone (P), total testosterone (T), sex hormone-binding globulin (SHBG), and anti-Müllerian hormone (AMH) were measured by electrochemiluminescence immunoassays (Roche Cobas, Germany). Free testosterone (FT) was calculated using Vermeulen' s formula, and the free androgen index (FAI) was calculated as (total testosterone × 100 / SHBG). FBG and fasting insulin (FIN) were measured by the glucose oxidase method and chemiluminescence, respectively. Homeostasis model assessment of insulin resistance (HOMA-IR) was computed as [FBG (mmol/L) × FIN (μIU/ml)] / 22.5. Lipid profiles, including total cholesterol (TC), TG, high-density lipoprotein cholesterol (HDL-C), low-density lipoprotein cholesterol (LDL-C), apolipoprotein A1 (APOA1), and apolipoprotein B (APOB), were measured using an automatic biochemical analyzer. Liver and kidney function parameters [AST, ALT, lactate dehydrogenase (LDH), urea (URE), creatinine, total bile acids (TBA) and total bilirubin (TBil)] were determined by standard enzymatic methods. The ZJU index was then calculated by combining BMI, FBG, TG, ALT, and AST levels using the formula: ZJU Index = BMI (kg/m2) + FBG (mmol/L) + TG (mmol/L) + 3 × (ALT/AST ratio) + 2 (for women) (12).

#### 2.2.2 Pregnancy outcome

Reproductive outcomes were defined as follows: ovulation-serum progesterone 3 ng/mL (however cutoffs may vary from lab to lab) per cycle; conception-serum hCG >10 IU/L (triggering intervention cessation); clinical pregnancy-intrauterine gestation with fetal cardiac activity confirmed by transvaginal ultrasound; pregnancy loss-including spontaneous abortion (< 20 weeks), fetal death (≥20 weeks), or stillbirth (≥20 weeks); and live birth-delivery of a viable neonate at ≥20 weeks of gestation.

### 2.3 Statistical analyses

All analyses were conducted using R software (version 4.5.0; R Foundation for Statistical Computing, Vienna, Austria), with two-tailed *p*-values < 0.05 considered statistically significant. To assess the missing data mechanism, the TestMCARNormality function from the MissMech package was applied to the main variables. Baseline characteristics were also compared between participants with complete vs. incomplete data. Because the data did not satisfy the multivariate normality assumption (Hawkins test *p* < 0.001), non-parametric test results were primarily relied upon (Supplementary material). In descriptive analyses, participants' baseline characteristics were stratified according to ZJU tertiles. The tertile cut-offs were 32.07 and 36.69, defining Q1 as ZJU ≤ 32.07, Q2 as 32.07 < ZJU ≤ 36.69, and Q3 as ZJU > 36.69. Continuous variables are presented as median (25th, 75th percentiles) and compared using the Kruskal–Wallis test. Categorical variables were summarized as counts (percentages) and compared using chi-square tests or Fisher's exact tests when expected frequencies were less than 5. Logistic regression models were used to evaluate the associations between ZJU and reproductive outcomes, including ovulation rate, conception rate, clinical pregnancy rate, and live birth rate. Multicollinearity was assessed using VIFs. The ZJU index was modeled both as a categorical variable (tertiles) and as a continuous variable. For tertile analyses, odds ratios (ORs) and 95% confidence intervals (CIs) were estimated using the lowest tertile as the reference. Tests for linear trend were conducted by modeling the median value of each tertile as a continuous variable. Model 1 was unadjusted. Model 2 was adjusted for interventions, age, SBP and DBP. Model 3, additionally including metabolic and hormonal covariates (FAI, SHBG, HOMAIR, P, TBA, TBIL, FT, Creatinine, LDH, AMH, LH, FSH), was fitted as a sensitivity analysis. Notably, WC and BMI were not included in the covariate adjustment due to severe collinearity with ZJU. Potential non-linear associations were evaluated using restricted cubic spline (RCS) analyses with four knots. The RCS analyses were repeated after excluding observations below the 1st percentile or above the 99th percentile of the ZJU index. E-values were calculated for the Model 2 odds ratios and the corresponding 95% confidence-interval limits closest to the null value of 1. To assess whether the effect of ZJU on reproductive outcomes differs according to androgen levels, we stratified participants by the median of FAI. An interaction term (ZJU × FAI group) was included in the logistic regression models, adjusted for the same covariates as in Model 2. Stratum-specific odds ratios were also estimated. Intervention assignment was included as a four-level categorical covariate. Exploratory treatment-stratified analyses and ZJU by treatment interaction tests were also performed.

## 3 Results

### 3.1 Baseline characteristics of the study population according to ZJU tertiles

Table 1 summarizes the baseline characteristics of participants stratified by ZJU tertiles (Q1, *n* = 310, Q2, *n* = 309, and Q3, *n* = 309). Higher ZJU tertiles were associated with progressively worse anthropometric, metabolic, and hormonal profiles. Women in higher ZJU tertiles showed significantly higher age, BMI, SBP, and DBP (all *P* < 0.05). Regarding sex hormone profiles, higher ZJU tertiles were associated with increased FT and FAI, and decreased E2, SHBG, and AMH (all *P* < 0.01). Regarding glycolipid metabolism, significant progressive increases were observed in FIN, FBG, HOMA-IR, LDL, TC, TG, and ApoB (all *P* < 0.001), whereas HDL and ApoA1 decreased (both *P* < 0.001). Regarding liver-kidney function profiles, significant progressive increases were observed in AST, ALT, and LDH, while creatinine, TBA, and TBil decreased (all *P* < 0.05). No significant differences were observed across ZJU tertiles in T, P, FSH, URE, and interventions. A clear inverse trend was observed between ZJU tertiles and reproductive success. The rates of conception (*P* = 0.036), clinical pregnancy (*P* = 0.004), live birth (*P* = 0.004), and ovulation (*P* < 0.001) were significantly higher in Q1 than in Q2 or Q3.

**Table 1:** Baseline characteristics of study population based on ZJU tertiles.

Characteristic	Q1 (*N* = 310, ZJU ≤ 32.07)	Q2 (*N* = 309, 32.07 < ZJU ≤ 36.69)	Q3 (*N* = 309, ZJU > 36.69)	*p*
ZJU index	29.69 (28.19, 30.79)	34.20 (33.12, 35.37)	39.74 (38.30, 42.54)	**< 0.001**
Anthropometric measures				
Age, years	27.00 (26.00, 29.00)	28.00 (26.00, 30.00)	28.00 (26.00, 30.00)	**0.0029**
BMI, kg/m^2^	20.18 (18.90, 21.30)	23.73 (22.50, 24.96)	28.03 (26.37, 30.37)	**< 0.001**
SBP, mmHg	110.00 (102.00, 115.00)	110.00 (110.00, 120.00)	115.00 (110.00, 120.00)	**< 0.001**
DBP, mmHg	70.00 (70.00, 80.00)	75.00 (70.00, 80.00)	76.00 (70.00, 80.00)	**< 0.001**
Sex hormone profiles				
P, nmol/L	1.80 (1.29, 2.46)	1.73 (1.19, 2.40)	1.68 (1.17, 2.27)	0.1188
T, nmol/L	1.50 (1.17, 1.99)	1.60 (1.18, 2.09)	1.64 (1.30, 2.04)	0.1357
FAI	2.74 (1.85, 4.98)	4.53 (2.85, 7.63)	7.13 (4.66, 10.34)	**< 0.001**
LH, mIU/ml	10.62 (7.25, 16.62)	9.88 (5.96, 14.28)	8.55 (5.20, 11.84)	**< 0.001**
FSH, mIU/ml	6.17 (5.17, 7.14)	5.99 (5.08, 7.01)	5.83 (4.98, 6.92)	0.0528
E2, pmol/L	208.90 (168.00, 278.90)	200.20 (163.45, 267.80)	187.00 (150.50, 242.10)	**< 0.001**
SHBG, nmol/L	51.65 (35.95, 72.88)	33.60 (23.15, 50.70)	22.70 (17.00, 32.35)	**< 0.001**
FT, pg/ml	2.05 (1.51, 2.63)	2.18 (1.67, 2.88)	2.38 (1.86, 2.91)	**< 0.001**
AMH, ng/ml	12.70 (8.29, 17.32)	11.65 (7.36, 15.79)	9.87 (6.30, 14.74)	**< 0.001**
Glycolipid metabolic profiles				
HOMA-IR	1.28 (0.84, 1.92)	2.37 (1.66, 3.39)	4.05 (2.86, 5.88)	**< 0.001**
FIN, μIU/ml	6.24 (4.10, 8.98)	10.63 (7.59, 14.75)	17.24 (12.79, 23.81)	**< 0.001**
FBG, mmol/L	4.75 (4.27, 5.19)	5.04 (4.61, 5.51)	5.33 (4.87, 5.86)	**< 0.001**
TC, mmol/L	4.40 (3.78, 5.00)	4.69 (3.98, 5.32)	5.02 (4.24, 5.79)	**< 0.001**
TG, mmol/L	0.96 (0.73, 1.18)	1.39 (1.00, 1.89)	1.93 (1.37, 2.72)	**< 0.001**
APOA1, g/L	1.57 (1.34, 1.78)	1.45 (1.28, 1.69)	1.46 (1.26, 1.64)	**< 0.001**
APOB, g/L	0.71 (0.60, 0.86)	0.87 (0.71, 1.05)	1.02 (0.84, 1.25)	**< 0.001**
HDL, mmol/L	1.40 (1.16, 1.63)	1.21 (1.03, 1.43)	1.13 (0.93, 1.35)	**< 0.001**
LDL, mmol/L	2.63 (2.14, 3.11)	2.96 (2.40, 3.47)	3.21 (2.56, 3.81)	**< 0.001**
Liver-kidney function profiles				
AST, U/L	11.00 (9.00, 14.00)	12.00 (9.00, 14.00)	13.00 (10.00, 17.00)	**< 0.001**
ALT, U/L	5.00 (3.00, 7.00)	6.00 (4.00, 9.00)	10.00 (6.00, 16.00)	**< 0.001**
LDH, U/L	76.00 (48.00, 107.00)	87.00 (50.00, 119.00)	91.00 (54.00, 124.00)	**0.0024**
URE, mmol/L	4.19 (3.55, 4.94)	4.17 (3.47, 5.00)	4.32 (3.62, 5.24)	0.154
Creatinine, μmol/L	42.65 (36.42, 50.48)	41.90 (35.00, 48.92)	40.70 (34.20, 49.10)	**0.0301**
Total bile acids (μ*mol*/*L*)	1.50 (0.80, 2.70)	1.25 (0.60, 2.20)	1.20 (0.60, 2.10)	**< 0.001**
Total bilirubin (μ*mol*/*L*)	6.35 (4.80, 9.17)	5.70 (4.10, 7.73)	5.10 (3.90, 6.80)	**< 0.001**
Fertility outcomes				
Conception	113 (36.5%)	101 (32.7%)	83 (26.9%)	**0.036**
Clinical pregnancy	85 (27.4%)	64 (20.7%)	51 (16.5%)	**0.004**
Live birth	81 (26.1%)	60 (19.4%)	48 (15.5%)	**0.004**
Ovulation	257 (82.9%)	252(81.6%)	218 (70.6%)	**< 0.001**
Interventions				0.4428
A: acupuncture + clomiphene	87 (28.1%)	71 (23.0%)	72 (23.3%)	
B: control acupuncture + clomiphene	74 (23.9%)	88 (28.5%)	73 (23.6%)	
C: acupuncture + placebo	80 (25.8%)	72 (23.3%)	81 (26.2%)	
D: control acupuncture + placebo	69 (22.3%)	78 (25.2%)	83 (26.9%)	

Bold values indicate statistical significance (*P* < 0.05).

### 3.2 Association between the ZJU index and reproductive outcomes

The associations between the ZJU index and reproductive outcomes (ovulation, conception, clinical pregnancy, and live birth) are summarized in Table 2. Variance inflation factors (VIFs) for all covariates were below 5, indicating no significant multicollinearity. In tertile analyses, higher ZJU tertiles were generally associated with lower odds of ovulation (OR = 0.53, 95% CI: 0.34–0.81), conception (OR = 0.68, 95% CI: 0.47–0.98), clinical pregnancy (OR = 0.56, 95% CI: 0.37–0.84), and live birth (OR = 0.58, 95% CI: 0.38–0.88), and the overall trends across tertiles were statistically significant in both unadjusted and adjusted models (*P* for trend < 0.05). When analyzed as a continuous variable, each one-unit increase in the ZJU index was associated with reduced odds of ovulation (OR = 0.94, 95% CI: 0.91–0.97), conception (OR = 0.96, 95% CI: 0.93–0.99), clinical pregnancy (OR = 0.95, 95% CI: 0.92–0.98), and live birth (OR = 0.95, 95% CI: 0.92–0.99) (all *P* < 0.05).

**Table 2:** ZJU by tertiles and association with reproductive outcomes.

Ovulation	*N* (%)	Model 1	Model 2	Conception	*N* (%)	Model 1	Model 2
ZJU (tertiles)				ZJU (tertiles)			
Q1	310 (33.4)	Reference	Reference	Q1	310 (33.4)		
Q2	309 (33.3)	0.91 (0.60–1.38)	0.94 (0.60–1.46)	Q2	309 (33.3)	0.85 (0.61–1.18)	0.87 (0.61–1.23)
*P*		0.661	0.785	*P*		0.325	0.427
Q3	309 (33.3)	0.49 (0.34–0.72)	0.53 (0.34–0.81)	Q3	309 (33.3)	0.64 (0.45–0.90)	0.68 (0.47–0.98)
*P*		< 0.001	0.003	*P*		0.011	0.037
*P* for trend		< 0.001	0.002	*P* for trend		0.011	0.037
Per 1 unit increase		0.93 (0.91–0.96)	0.94 (0.91–0.97)	Per 1 unit increase		0.96 (0.93–0.98)	0.96 (0.93–0.99)
*P*		< 0.001	< 0.001	*P*		< 0.001	0.004
Clinical pregnancy		Model 1	Model 2	Live birth		Model 1	Model 2
ZJU (tertiles)				ZJU (tertiles)			
Q1	310 (33.4)	Reference	Reference	Q1	310 (33.4)	Reference	Reference
Q2	309 (33.3)	0.69 (0.48–1.00)	0.71 (0.48–1.04)	Q2	309 (33.3)	0.68(0.47–0.99)	0.72 (0.49–1.07)
*P*		0.0516	0.081	*P*		0.047	0.102
Q3	309 (33.3)	0.52 (0.35–0.77)	0.56 (0.37–0.84)	Q3	309 (33.3)	0.52 (0.35–0.77)	0.58 (0.38–0.88)
*P*		0.001	0.006	*P*		0.001	0.012
*P* for trend		0.001	0.005	*P* for trend		0.001	0.011
Per 1 unit increase		0.95(0.92–0.98)	0.95 (0.92–0.98)	Per 1 unit increase		0.94 (0.91–0.98)	0.95 (0.92–0.99)
*P*		< 0.001	0.002	*P*		< 0.001	0.006

Model 1: non–adjusted.

Model 2: adjusted for interventions, age, SBP, and DBP.

### 3.3 Detection of nonlinear relationships

**Figure 2:**
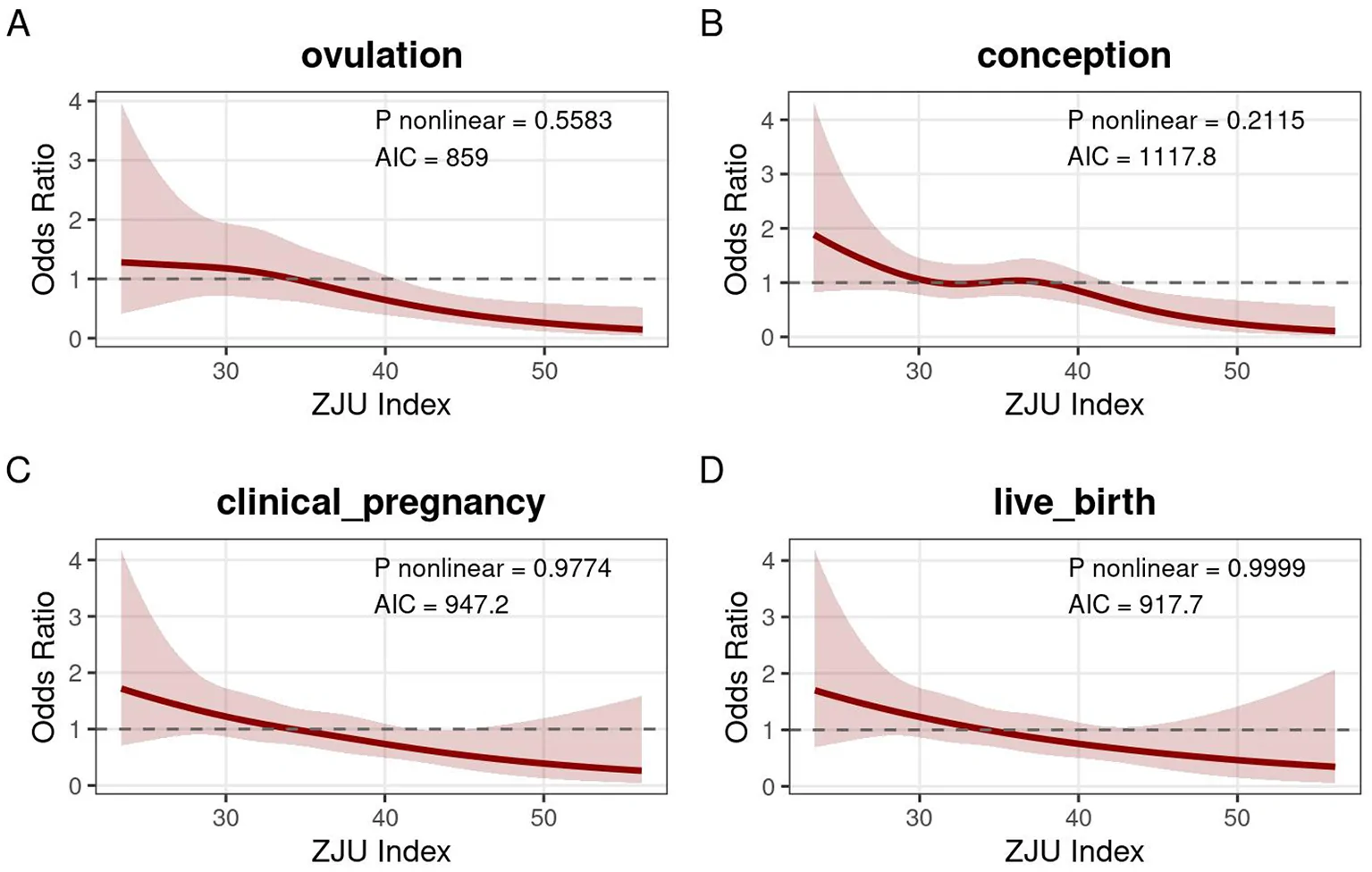
Non-linear relationship between ZJU and ovulation **(A)**, conception **(B)**, clinical pregnancy **(C)**, and live birth **(D)**. The OR and 95% CI were derived from the logistic regression model adjusted for interventions, age, SBP, and DBP, with the reference point being the median of ZJU. The red solid line represents the OR curve, the red translucent band represents the 95% confidence interval, and the gray dotted line represents OR = 1. The nonlinear test *P* value and AIC are labeled in the upper right corner of each subgraph.

To better characterize the relationship between ZJU and reproductive outcomes, RCS analyses based on logistic regression models were conducted (Figure 2). After adjusting for the same covariates as in Model 2, no significant nonlinear relationships were observed for ovulation (*P* for nonlinearity = 0.558), conception (*P* for nonlinearity = 0.212), clinical pregnancy (*P* for nonlinearity = 0.977), or live birth (*P* for nonlinearity = 0.999).

### 3.4 Sensitivity analyses

Data missingness did not deviate from the MCAR assumption (*P* = 0.578, non-parametric MCAR test). Furthermore, participants with complete and incomplete records had comparable baseline features, making it unlikely that missing data were strongly associated with the exposure or confounders (Supplementary Table S2). When we restricted the primary models to those with full data for all Model 3 covariates (*N* = 896), the findings remained virtually unchanged (Supplementary Table S3; Supplementary Figure S1). After excluding extreme ZJU values, evidence of nonlinearity was observed for ovulation (*P* = 0.029), but not for conception, clinical pregnancy, or live birth (Supplementary Figure S2). For the continuous ZJU analysis, the E-values for the point estimate and the 95% confidence-interval limit closest to the null were 1.22 and 1.15 for ovulation, 1.17 and 1.09 for conception, 1.19 and 1.11 for clinical pregnancy, and 1.18 and 1.09 for live birth, respectively. For Q3 vs. Q1, the corresponding E-values were 2.10 and 1.47, 1.73 and 1.12, 2.01 and 1.40, and 1.95 and 1.32, respectively (Supplementary Table 4). The relatively modest confidence-limit E-values indicate that residual confounding cannot be excluded. In exploratory treatment-stratified analyses, baseline ZJU was balanced across treatment groups (*P* = 0.368 for continuous ZJU and *P* = 0.443 for ZJU tertiles). No significant ZJU-by-treatment interaction was observed for conception, clinical pregnancy, or live birth (*P* for interaction = 0.257, 0.160, and 0.149, respectively), whereas a nominal interaction was observed for ovulation (*P* for interaction = 0.047; Supplementary Table 5). This finding should be interpreted cautiously because the subgroup analysis was exploratory.

### 3.5 Subgroup analysis

Participants were stratified by the median FAI value (4.735) into high and low FAI groups. The associations between the ZJU index and reproductive outcomes were examined within each stratum, and interaction terms were tested. Although none of the interaction terms reached statistical significance (all *P* > 0.05), the inverse associations of the ZJU index with clinical pregnancy and live birth were significant only among women with higher FAI, suggesting a possible effect modification by androgen status (Table 3).

**Table 3:** Subgroup analysis: stratify by FAI categories and interaction effects for the association between the ZJU and reproductive outcomes.

Outcome	FAI categories	OR (95% CI)	*P*	*P* for interaction
Ovulation				0.7142
	FAI ≥ 4.735	0.96 (0.92–1.00)	0.0476	
	FAI < 4.735	0.95 (0.89–1.01)	0.0773	
Conception				0.5105
	FAI ≥ 4.735	0.96 (0.91–1.00)	0.0707	
	FAI < 4.735	0.98 (0.94–1.03)	0.4067	
Clinical pregnancy				0.2543
	FAI ≥ 4.735	0.93 (0.88–0.98)	0.0135	
	FAI < 4.735	0.98 (0.93–1.03)	0.4144	
Live birth				0.2417
	FAI ≥ 4.735	0.94 (0.88–0.99)	0.0223	
	FAI < 4.735	0.98 (0.93–1.03)	0.4896	

Adjusted factors are the same as those in model 2.

## 4 Discussion

This secondary analysis of the PCOSAct trial demonstrates that a higher baseline ZJU index is independently and linearly inversely associated with the likelihood of ovulation (OR = 0.94, 95% CI: 0.91–0.97), conception (OR = 0.96, 95% CI: 0.93–0.99), clinical pregnancy (OR = 0.95, 95% CI: 0.92–0.98), and live birth (OR = 0.95, 95% CI: 0.92–0.99) among anovulatory women with PMOS undergoing standardized fertility treatment. Sensitivity analyses showed generally consistent directions, although some estimates were attenuated after extended adjustment. To our knowledge, this is the first investigation to link the ZJU index directly to prospectively collected reproductive treatment endpoints in women with PMOS, thereby extending prior cross-sectional evidence that the index correlates with infertility risk in the general population (13) and with PMOS diagnosis among women undergoing assisted reproduction (16).

The ZJU index was originally developed and validated as a screening tool for NAFLD in the Chinese population, demonstrating superior diagnostic accuracy (0.925) compared to other indices (10). Subsequent studies have expanded its utility: Ji et al. showed that the ZJU index is a useful indicator of insulin resistance in the general Chinese population (12). Zheng et al. reported that higher ZJU index predicts new-onset NAFLD in non-obese individuals with a hazard ratio of 21.67 (11). And Tian et al. found a positive association between ZJU and infertility risk in reproductive-aged US women (OR = 1.04) (13). Most recently, Yin et al. demonstrated that the ZJU index is associated with PMOS diagnosis, with serum total testosterone mediating 14.5% of the effect (16). This study is consistent with our findings. When we stratified by FAI, the inverse associations with clinical pregnancy and live birth were statistically significant only among women with higher FAI (≥4.735). It suggests that hyperandrogenemia may amplify the reproductive toxicity of metabolic dysregulation. Our study builds directly on this evidence by showing that, among women with established PMOS and anovulatory infertility, a higher ZJU index was associated with poorer outcomes during fertility treatment, moving from association with diagnosis to association with treatment success.

Consistent with the pathophysiology of PMOS, in which IR and hyperinsulinemia drive both metabolic and reproductive disturbances (17), our data suggest that the ZJU index effectively captures the cumulative burden of these comorbidities. These abnormalities can disrupt folliculogenesis, impair endometrial receptivity (18), and compromise placental vascular remodeling, thereby leading to anovulation, subfertility, and early pregnancy loss. Our finding that each one-unit increase in ZJU reduced the odds of live birth by approximately 5% suggests that the index captures a cumulative metabolic burden that conventional single parameter markers miss. Importantly, the ZJU index has also been shown to predict gestational diabetes mellitus (OR = 1.22, AUC = 0.802) (19) and cardiovascular disease (20), further supporting its broad metabolic relevance.

Obesity is a central driver of adverse reproductive outcomes in women with PMOS. Over 50% of women with PMOS are overweight or obese, and adipose tissue dysfunction in this population, characterized by adipocyte hypertrophy, chronic low-grade inflammation, and impaired insulin signaling exacerbates both IR and HA, creating a vicious cycle that impairs folliculogenesis and endometrial receptivity (21, 22). Large population based cohort studies have confirmed that overweight and obesity independently increase the risk of adverse pregnancy outcomes (23), and this association is similarly pronounced among PMOS patients undergoing assisted reproduction (24). Elevated maternal BMI has been consistently linked to reduced clinical pregnancy and live birth rates after embryo transfer (25, 26). Among patients undergoing *in vitro* fertilization, Obese women have been specifically linked to reduced rates of clinical pregnancy (OR = 0.69, 95% CI: 0.52–0.93) and live birth (OR = 0.58, 95% CI: 0.43–0.80), even when standardized blastocyst transfer protocols are employed (26). Importantly, weight reduction can effectively restore ovulatory function. A multicenter randomized controlled trial demonstrated that bariatric surgery induces spontaneous ovulation in women with PMOS, providing robust evidence that substantial weight loss can restore ovulatory function (27). Mechanistically, obesity compromises fertility through multiple pathways. Adipose tissue derived proinflammatory cytokines disrupt the follicular microenvironment and the endometrial implantation window, and immune cell imbalance has been documented in the follicular fluid of overweight and obese women (28). Furthermore, obesity induced glycolipid dysmetabolism can be mediated by the systemic immune inflammation index to further compromise IVF/ICSI outcomes (29).

Dyslipidemia constitutes a key component of the metabolic disturbance spectrum in PMOS. Recent evidence confirms that dyslipidemia exerts an independent adverse effect on embryo quality (30, 31). In patients undergoing their first IVF/ICSI cycle, lipid metabolism abnormalities significantly reduce the rates of good quality embryos and cumulative live birth (32). In particular, a slightly elevated triglyceride level significantly decreases the oocyte retrieval rate compared to a normal triglyceride level (OR = 0.950, 95% CI: 0.912–0.988). Elevated TG not only directly reflect increased hepatic lipid synthesis under IR conditions but may also impair oocyte quality and early embryonic developmental potential through lipotoxicity (33). The inclusion of triglycerides therefore may capture additional metabolic information of the ZJU index for reproductive outcomes. Once obesity is established, IR becomes an almost inevitable companion, and FBG serves as the most direct window into this pathological process. Indeed, studies have observed that even among women with normal BMI, high normal FBG levels have been associated with reduced live birth rates (OR = 0.747, 95% CI: 0.541–0.963) following frozen thawed single blastocyst transfer (34). This effect extends to male partners as well, where high normal fasting glucose adversely affects semen quality and embryonic development (35). Given that IR and impaired glucose tolerance are highly prevalent in PMOS, FBG enables the capture of subclinical glycemic dysregulation that BMI alone cannot fully reflect, allowing it to identify high risk patients whose body weight remains within the normal range but who have already developed early glycemic dysregulation.

The impact of IR on the liver is equally profound, and both TG and liver enzymes serve as serological manifestations of this burden. Evidence has demonstrated that a higher baseline ALT level is independently associated with a lower live birth rate after freeze thawed embryo transfer (36). In the PMOS population, the high prevalence of nonalcoholic fatty liver disease further amplifies the potential impact of liver enzyme abnormalities on reproductive prognosis. The inclusion of the ALT to AST ratio thus allows the ZJU index to reflect the crosstalk between the liver and the reproductive axis that conventional metabolic markers fail to capture. The ZJU index captures IR, which directly impairs folliculogenesis, oocyte quality, and endometrial receptivity (18, 37). IR driven hyperinsulinemia stimulates ovarian androgen production, exacerbating hyperandrogenism and further disrupting ovulation (5). The inclusion of triglycerides and the ALT/AST ratio may reflect dyslipidemia and hepatic steatosis, both of which are highly prevalent in PMOS and associated with chronic low grade inflammation and oxidative stress (17, 22). This inflammatory environment can compromise oocyte developmental competence and implantation potential. Collectively, these pathways provide a coherent mechanistic explanation for why a higher ZJU index, reflecting a greater cumulative metabolic burden, was associated with poorer reproductive outcomes.

We innovatively investigated the association between the ZJU index and the reproductive outcomes in PMOS patients. It requires only routine blood tests and BMI, all of which are already collected in most infertility workups. The calculation is simple and does not require specialized software. Although higher ZJU values were associated with lower odds of live birth, an association measure should not be interpreted as evidence of adequate individual-level predictive performance. Future studies should evaluate whether the ZJU index adds incremental predictive value to established reproductive factors in externally validated multivariable prediction models. Given that the ZJU index has already been shown to predict GDM and NAFLD, its utility may extend beyond fertility outcomes to pregnancy complications. However, several limitations warrant consideration. First, as a secondary analysis of an existing dataset, we are restricted to the variables originally collected. Although we adjusted for intervention assignments, we cannot exclude residual confounding from unmeasured factors such as diet, physical activity, or genetics. Second, the study population was entirely Chinese, all receiving acupuncture and/or clomiphene; generalizability to other ethnic groups or other treatment protocols remains unknown. The nominal ZJU-by-treatment interaction for ovulation was exploratory and should be interpreted cautiously. Third, the ZJU index was originally developed for NAFLD screening; its use in reproductive medicine is novel, and cross validation in non-Asian populations is needed. Fourth, the modest E-values, particularly those for the confidence limits, indicate that residual confounding from unmeasured lifestyle, socioeconomic, phenotypic, or genetic factors cannot be excluded. Therefore, these associations should not be interpreted as causal. Fifth, the cross sectional assessment of baseline ZJU precludes conclusions about how changes in ZJU over time might affect outcomes.

## 5 Conclusion

A higher baseline ZJU index is independently associated with lower rates of ovulation, conception, clinical pregnancy, and live birth in Chinese women with PMOS undergoing fertility treatment. The index integrates multiple metabolic dimensions that BMI or single biomarkers alone cannot capture. Our findings align with a growing body of evidence positioning the ZJU index as a simple, low cost tool for metabolic risk stratification. Further prospective validation in diverse populations is warranted to establish its clinical utility.

## Data Availability

The raw data supporting the conclusions of this article will be made available by the authors, without undue reservation.
