# Supplementary material for: Associations of the Zhejiang University (ZJU) index with reproductive outcomes in women with polyendocrine metabolic ovarian syndrome: a post-hoc analysis
Source: Front Nutr. 2026 Aug 24;13:1892813. doi: 10.3389/fnut.2026.1892813 (PMC13548800; doi:10.3389/fnut.2026.1892813)
Supplement: Supplementary file 1 [file Data_Sheet_1.docx]

**Supplementary material**

**Supplementary Table 1. Missing rate of variables.**

| **variables** | **Unmissing (n)** | **Missing** (n) | **Missing proportion**% | **variables** | **Unmissing (n)** | **Missing** (n) | **Missing proportion**% |
| --- | --- | --- | --- | --- | --- | --- | --- |
| Age | 928 | 0 | 0 | TG | 928 | 0 | 0 |
| BMI | 928 | 0 | 0 | APOA1 | 928 | 0 | 0 |
| SBP | 928 | 0 | 0 | APOB | 927 | 1 | 0.11 |
| DBP | 928 | 0 | 0 | HDL | 926 | 2 | 0.22 |
| P | 921 | 7 | 0.75 | LDL | 927 | 1 | 0.11 |
| T | 924 | 4 | 0.43 | ALT | 928 | 0 | 0 |
| FAI | 916 | 12 | 1.29 | AST | 928 | 0 | 0 |
| LH | 922 | 6 | 0.65 | LDH | 926 | 2 | 0.22 |
| FSH | 923 | 5 | 0.54 | URE | 927 | 1 | 0.11 |
| LH/FSH | 923 | 5 | 0.54 | Creatinine | 927 | 1 | 0.11 |
| E2 | 923 | 5 | 0.54 | TBA | 921 | 7 | 0.75 |
| SHBG | 916 | 12 | 1.29 | TBIL | 927 | 1 | 0.11 |
| FT | 923 | 5 | 0.54 | Interventions | 928 | 0 | 0 |
| AMH | 928 | 0 | 0 | conception | 928 | 0 | 0 |
| HOMAIR | 923 | 5 | 0.54 | clinical pregnancy | 928 | 0 | 0 |
| FIN | 923 | 5 | 0.54 | live birth | 928 | 0 | 0 |
| FBG | 928 | 0 | 0 | ovulation | 928 | 0 | 0 |
| TC | 927 | 1 | 0.11 |  |  |  |  |

**Supplementary Table 2. Baseline characteristics according to missing data status (based on Model 3, complete-case vs incomplete)**

| Variable | Complete  N=896 | Incomplete  N=32 | *P* |
| --- | --- | --- | --- |
| ZJU | 34.78 (5.33) | 35.35 (5.17) | 0.5452 |
| Age | 27.91 ± 3.32 | 28.06 ± 2.93 | 0.7777 |
| BMI | 24.15 ± 4.22 | 24.49 ± 4.65 | 0.69 |
| SBP | 112.33 ± 9.35 | 111.66 ± 8.31 | 0.657 |
| DBP | 74.89 ± 7.83 | 74.28 ± 8.29 | 0.6834 |
| P | 1.73 (1.21-2.40) | 1.55 (1.30-2.05) | 0.6756 |
| T | 1.59 (1.20-2.04) | 1.53 (1.30-1.95) | 0.8542 |
| FAI | 4.75 (2.58-7.72) | 4.17 (2.12-9.06) | 0.937 |
| LH | 9.41 (6.25-14.19) | 8.21 (4.87-12.86) | 0.2294 |
| FSH | 6.01 (5.06-7.05) | 6.23 (5.02-7.05) | 0.8053 |
| E2 | 198.45 (158.88-264.57) | 204.80 (168.00-244.05) | 0.6862 |
| SHBG | 34.00 (21.87-54.80) | 31.20 (23.00-62.38) | 0.7431 |
| FT | 2.21 (1.67-2.79) | 1.90 (1.46-2.91) | 0.3803 |
| AMH | 12.12 ± 6.43 | 12.31 ± 4.50 | 0.8132 |
| HOMAIR | 2.31 (1.42-3.83) | 2.29 (1.56-3.38) | 0.9819 |
| FIN | 10.58 (6.83-16.70) | 10.84 (6.78-15.89) | 0.9436 |
| FBG | 5.04 ± 0.94 | 5.18 ± 1.37 | 0.5544 |
| TC | 4.74 ± 1.08 | 5.02 ± 1.11 | 0.1722 |
| TG | 1.56 ± 0.90 | 1.84 ± 0.92 | 0.0949 |
| APOA1 | 1.51 ± 0.32 | 1.50 ± 0.30 | 0.7831 |
| APOB | 0.90 ± 0.28 | 1.00 ± 0.30 | 0.0676 |
| HDL | 1.29 ± 0.37 | 1.18 ± 0.36 | 0.1174 |
| LDL | 2.97 ± 0.88 | 3.07 ± 0.80 | 0.5013 |
| ALT | 8.99 ± 8.68 | 9.22 ± 6.01 | 0.8366 |
| AST | 13.00 ± 7.33 | 14.97 ± 7.72 | 0.1648 |
| LDH | 84.00 (50.00-116.00) | 102.50 (53.50-126.75) | 0.3699 |
| URE | 4.38 ± 1.28 | 4.60 ± 1.27 | 0.3462 |
| Creatinine | 42.85 ± 10.66 | 46.55 ± 13.70 | 0.1471 |
| TBA | 1.30 (0.70-2.40) | 1.40 (1.20-2.10) | 0.6064 |
| TBIL | 6.40 ± 3.17 | 6.22 ± 3.11 | 0.758 |
| ovulation | 705 (78.7%) | 22 (68.8%) | 0.2619 |
| conception | 286 (31.9%) | 11 (34.4%) | 0.9206 |
| clinical_pregnancy | 194 (21.7%) | 6 (18.8%) | 0.8623 |
| live_birth | 183 (20.4%) | 6 (18.8%) | 0.9939 |
| Interventions |  |  |  |
| Acupuncture + clomiphene | 225 (25.1%) | 5 (15.6%) | 0.2062 |
| Control acupuncture + clomiphene | 228 (25.4%) | 7 (21.9%) |  |
| Acupuncture + placebo | 220 (24.6%) | 13 (40.6%) |  |
| Control acupuncture + placebo | 223 (24.9%) | 7 (21.9%) |  |
| To assess the mechanism of missing data, we used the `TestMCARNormality` function from the `MissMech` package to perform an MCAR test on the main continuous variables. Since the data did not satisfy the assumption of multivariate normality (Hawkins test, P < 0.001), we relied primarily on the results of the nonparametric test. The P-value for the non-parametric test was 0.578, indicating that no significant evidence was found to reject the hypothesis of completely random missing data (MCAR). The nonparametric test did not reject the MCAR hypothesis (*P*=0.578), indicating no evidence that the missingness mechanism departed from MCAR. | | | |

| **Supplementary Table 3. Sensitivity Analysis: Association between ZJU and** **reproductive outcomes (N=896).** | | | | | | | | | |
| --- | --- | --- | --- | --- | --- | --- | --- | --- | --- |
| Ovulation | N (%) | Model 1 | Model 2 | Model3 | Conception | N (%) | Model 1 | Model 2 | Model3 |
| ZJU (tertiles) |  |  |  |  | ZJU (tertiles) |  |  |  |  |
| Q1 | 299 (33.4) | Reference | Reference | Reference | Q1 | 299 (33.4) | Reference | Reference | Reference |
| Q2 | 298 (33.3) | 0.97 (0.64-1.48) | 1.02 (0.65-1.61) | 1.08 (0.65-1.79) | Q2 | 298 (33.3) | 0.84 (0.6-1.18) | 0.86 (0.60-1.22) | 0.97 (0.66-1.44) |
| *P* |  | 0.899 | 0.921 | 0.765 | *P* |  | 0.316 | 0.386 | 0.887 |
| Q3 | 299 (33.4) | 0.52 (0.35-0.77) | 0.57 (0.37-0.87) | 0.59 (0.33-1.05) | Q3 | 299 (33.4) | 0.64 (0.45-0.90) | 0.67(0.46-0.97) | 0.86 (0.53-1.39) |
| *P* |  | 0.001 | 0.011 | 0.072 | *P* |  | 0.011 | 0.035 | 0.538 |
| *P* for trend |  | <0.001 | 0.007 | 0.057 | *P* for trend |  | 0.011 | 0.035 | 0.54 |
| Per 1 unit increase |  | 0.93 (0.91-0.96) | 0.94 (0.91-0.97) | 0.92 (0.87-0.96) | Per 1 unit increase |  | 0.95 (0.93-0.98) | 0.95 (0.93-0.98) | 0.97 (0.93-1.00) |
| *P* |  | <0.001 | <0.001 | <0.001 | *P* |  | <0.001 | 0.002 | 0.081 |
| Clinical pregnancy |  | Model 1 | Model 2 | Model3 | Live birth |  | Model 1 | Model 2 | Model3 |
| ZJU (tertiles) |  |  |  |  | ZJU (tertiles) |  |  |  |  |
| Q1 | 299 (33.4) | Reference | Reference | Reference | Q1 | 299 (33.4) | Reference | Reference | Reference |
| Q2 | 298 (33.3) | 0.67 (0.46-0.98) | 0.69 (0.47-1.02) | 0.81 (0.52-1.25) | Q2 | 298 (33.3) | 0.66 (0.45-0.97) | 0.70 (0.47-1.04) | 0.86 (0.55-1.34) |
| *P* |  | 0.039 | 0.061 | 0.341 | *P* |  | 0.035 | 0.0784 | 0.508 |
| Q3 | 299 (33.4) | 0.49 (0.33-0.73) | 0.53 (0.34-0.80) | 0.76 (0.44-1.32) | Q3 | 299 (33.4) | 0.49(0.321-0.726) | 0.55 (0.35-0.84) | 0.88 (0.50-1.53) |
| *P* |  | <0.001 | 0.003 | 0.332 | *P* |  | <0.001 | 0.006 | 0.639 |
| *P* for trend |  | <0.001 | 0.003 | 0.318 | *P* for trend |  | <0.001 | <0.001 | 0.614 |
| Per 1 unit increase |  | 0.94 (0.91-0.97) | 0.94 (0.91-0.97) | 0.97 (0.92-1.01) | Per 1 unit increase |  | 0.94 (0.90-0.97) | 0.94 (0.91-0.98) | 0.98 (0.93-1.03) |
| *P* |  | <0.001 | <0.001 | 0.137 | *P* |  | <0.001 | 0.001 | 0.343 |
| Model1: Non-adjusted. | | | | | | | | | |
| Model2: Adjusted for interventions, age, SBP, and DBP. | | | | | | | | | |
| Model3: Adjusted for interventions, age, SBP, DBP, FAI, SHBG, HOMAIR, P, TBA, TBIL, FT, Creatinine, LDH, AMH, LH, FSH. | | | | | | | | | |

**Supplementary Figure 1. Sensitivity Analysis: RCS Curves (N = 896; Model 3)**

Adjusted for interventions, age, SBP, DBP,FAI, SHBG, HOMAIR, P, TBA, TBIL, FT, Creatinine, LDH, AMH, LH, FSH.

**Supplementary Figure 2. Sensitivity Analysis: RCS Curves (excluding the extreme values of ZJU)**

After excluding the extreme values of ZJU (values below the 1st percentile and the 99th percentile), the restricted cubic spline analysis of the ZJU index and reproductive outcomes was conducted.

**Supplementary Table 4. Sensitivity analysis for unmeasured confounding using E-values.**

| Outcome | Adjusted OR (95% CI) | Exposure contrast | E-value for the point estimate | E-value for the 95% CI limit closest to the null |
| --- | --- | --- | --- | --- |
| Ovulation | 0.94 (0.91-0.97) | Per 1-unit increase in ZJU | 1.22 | 1.15 |
|  | 0.53 (0.34–0.81) | Q3 versus Q1 | 2.10 | 1.47 |
| Conception | 0.96 (0.93-0.99) | Per 1-unit increase in ZJU | 1.17 | 1.09 |
|  | 0.68 (0.47–0.98) | Q3 versus Q1 | 1.73 | 1.12 |
| Clinical pregnancy | 0.95 (0.92-0.98) | Per 1-unit increase in ZJU | 1.19 | 1.11 |
|  | 0.56 (0.37–0.84) | Q3 versus Q1 | 2.01 | 1.40 |
| Live birth | 0.95 (0.92-0.99) | Per 1-unit increase in ZJU | 1.18 | 1.09 |
|  | 0.58 (0.38–0.88) | Q3 versus Q1 | 1.95 | 1.32 |
| ORs were obtained from Model 2. E-values were calculated from the exact, unrounded estimates using the common-outcome option (rare = FALSE) in the EValue R package. Because all ORs were below 1, the confidence limit closest to the null was the upper confidence limit. | | | | |

**Supplementary Table 5**. **Exploratory treatment-stratified associations between the ZJU index and reproductive outcome**

| **Outcomes** | **Group A, OR (95% CI)** | **Group B, OR (95% CI)** | **Group C, OR (95% CI)** | **Group D, OR (95% CI)** | ***P* for interaction** |
| --- | --- | --- | --- | --- | --- |
| Ovulation | 0.90 (0.82–1.00) | 0.86 (0.79–0.94) | 0.97 (0.92–1.02) | 0.94 (0.89–0.99) | 0.047 |
| Conception | 1.00 (0.95–1.06) | 0.96 (0.91–1.02) | 0.92 (0.86–0.99) | 0.93 (0.87–0.99) | 0.257 |
| Clinical pregnancy | 1.00 (0.94–1.06) | 0.95 (0.90–1.02) | 0.88 (0.80–0.97) | 0.92 (0.85–0.99) | 0.160 |
| Live birth | 1.01 (0.95–1.07) | 0.95 (0.89–1.02) | 0.89 (0.81–0.98) | 0.92 (0.85–0.99) | 0.149 |
| ORs represent the change in the odds of each reproductive outcome per one-unit increase in the ZJU index and were adjusted for age, systolic blood pressure, and diastolic blood pressure within each treatment group. Group A: active acupuncture plus clomiphene; Group B: control acupuncture plus clomiphene; Group C: active acupuncture plus placebo; Group D: control acupuncture plus placebo. P values for interaction were obtained from likelihood-ratio tests of the joint ZJU-by-treatment interaction terms. | | | | | |
